# First Visit Fallout: Canadian Triage and Acuity Scale (CTAS) and Emergency Department Returns

**DOI:** 10.7759/cureus.82441

**Published:** 2025-04-17

**Authors:** David Lewis, Jacqueline Fraser, Michael Howlett, Matthew Greer, Paul Atkinson

**Affiliations:** 1 Emergency Medicine, Dalhousie Medicine New Brunswick, Saint John, CAN; 2 Emergency Medicine, Saint John Regional Hospital, Saint John, CAN; 3 Emergency Medicine, Dalhousie University, Halifax, CAN; 4 Emergency Medicine, Queen's University, Oshawa, CAN

**Keywords:** canadian triage and acuity scale, emergency department, hospital admission, patient safety, quality indicators, unplanned return visits

## Abstract

Introduction

Unplanned return visits (URVs) to the emergency department (ED) within 72 hours are an important quality indicator in emergency medicine, linked to patient safety and the quality of initial care. This study examines whether the Canadian Triage and Acuity Scale (CTAS) category at the initial visit predicts the likelihood of hospital admission upon URV.

Methods

A retrospective analysis was conducted over a 12-month period at a tertiary care teaching hospital. URVs were defined as registrations within 72 hours of an initial ED discharge, excluding planned returns. Data were extracted from electronic health records, including demographics, CTAS category, disposition, and admission status. Statistical analyses included Pearson correlation, linear regression, and Fisher’s exact test to examine relationships between CTAS and admission risk. Statistical significance was set at p < 0.05.

Results

Of 57,025 ED attendances, 82.1% (46,793) were discharged, of whom 7.6% (3,566) returned within 72 hours. Among URVs, 14.9% (532) resulted in admission. Admission rates on return varied by initial CTAS level, ranging from 23.1% (CTAS 1) to 4.8% (CTAS 5). CTAS 3 patients represented over half of all visits and the highest absolute number of return admissions. A strong negative correlation was observed between CTAS level and URV admission rate (Pearson r = -0.89; p = 0.04). Linear regression confirmed a statistically significant inverse trend, with each one-point increase in CTAS corresponding to a 5.4% absolute reduction in admission rate (R² = 0.90, p = 0.014). Patients triaged as CTAS 1-2 had a relative risk of 1.90 (95% CI: 1.57 to 2.30) for admission on return compared to those triaged as CTAS 3-5.

Conclusions

The initial CTAS level is a strong predictor of admission following URVs. Stratified analysis revealed that CTAS 3 patients comprise a clinically important group, both in volume and admission risk. These findings support the use of triage-based reporting in ED quality improvement initiatives.

## Introduction

Unplanned return visits (URVs) to the emergency department (ED) within 48-72 hours are widely recognized as a key quality indicator in emergency medicine [1]. A higher URV rate may reflect suboptimal discharge decisions, inadequate follow-up instructions, or evolving patient conditions. Among various factors influencing URVs, the Canadian Triage and Acuity Scale (CTAS) plays a pivotal role in prioritizing care based on clinical urgency [2].

International data exists regarding URVs, but there are inconsistencies in how URVs are defined and measured. Most studies, like ours, use 72 hours, but varying time periods are used internationally [3-6]. To our knowledge, the relationship between initial CTAS scores and outcomes of URVs has not been thoroughly explored in Canada [1-4]. There are no published Canadian data on the percentage of ED URV admissions.

While the CTAS is widely used to prioritize care in Canadian EDs, its relationship to downstream outcomes, such as hospital admission following an URV, is not fully understood. Understanding how admission rates vary by initial triage category may help inform quality improvement efforts and ED performance measurement.

This study aimed to describe URV rates and admission outcomes stratified by CTAS level at the initial ED visit, using retrospective data from a large academic ED. By reporting outcomes by triage category, this analysis offers insight into how initial acuity may relate to short-term clinical trajectory and guide system-level improvement strategies.

## Materials and methods

Study design and setting

This retrospective study was conducted at a Canadian 445-bed regional tertiary care adult and pediatric teaching hospital, which had 57,025 ED visits over the 12-month period under review. The study period spanned a single calendar year. In Canada, both ED and primary care visits are covered by the publicly funded healthcare system. Patients do not pay directly for these medical services.

Definition of URVs

URVs were defined as patient registrations within 72 hours of an earlier ED visit that had resulted in discharge. Planned return visits, such as follow-ups for wound care or diagnostic tests, were excluded.

Data collection

Data were extracted from the hospital’s electronic health records, including demographics, CTAS categories, disposition outcomes, and admission status. The primary outcome was hospital admission on URV. Secondary outcomes included overall URV rates and return/admission rates stratified by CTAS category.

Statistical analysis

Pearson correlation was used to assess the relationship between initial CTAS level and admission rates on return visits, treating CTAS as an ordinal variable (1-5). Fisher’s exact test was used to compare admission rates between grouped high-acuity (CTAS 1 and 2) and lower-acuity (CTAS 3, 4, and 5) categories, consistent with previous literature and reporting conventions.

For relative risk (RR) calculations, CTAS 4 was selected as the reference group due to its moderate acuity and large discharge population, providing a stable comparator. However, given the clinical differences among CTAS 3, 4, and 5, and the large volume of CTAS 3 patients, we also conducted and reported all analyses by individual CTAS level to allow for more granular interpretation.

A supplementary linear regression was performed, treating CTAS level as a continuous ordinal variable to assess trends in admission rate across acuity categories. Statistical significance was set at p < 0.05 for all analyses.

## Results

Overall URV and admission rates

**Figure 1:**
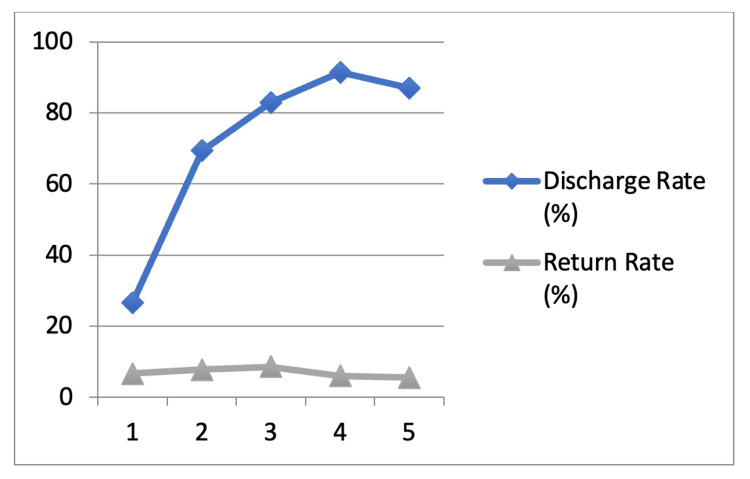
ED discharge rates (%) and URV rates (%) based on initial visit CTAS category CTAS: Canadian Triage and Acuity Scale; ED: emergency department; URV: unplanned return visit

Of 57,025 ED attendances, 1.3% (724) were triaged as CTAS 1, 18.0% (10,250) as CTAS 2, 52.5% (29,920) as CTAS 3, 25.9% (14,781) as CTAS 4, and 2.3% (1,284) as CTAS 5. An additional 0.1% (66) of patients had no triage classification recorded. Discharge and return rates by CTAS category are shown in Table 1 and Figure 1.

**Table 1:** Raw data for registrations, discharges, URV, and URV admissions CTAS: Canadian Triage and Acuity Scale; URV: unplanned return visit

CTAS level	Registrations	Discharged	Returned	Admitted on return
CTAS 1	724	193	13	3
CTAS 2	10,250	7,100	558	136
CTAS 3	29,920	24,801	2,118	335
CTAS 4	14,781	13,529	811	54
CTAS 5	1,284	1,117	62	3
No triage	66	53	4	1
Total	57,025	46,793	3,566	532

Overall, 82.1% (46,793) of patients were discharged from the ED. Discharge rates varied by CTAS category: 26.7% (193) for CTAS 1, 69.3% (7,100) for CTAS 2, 82.9% (24,801) for CTAS 3, 91.5% (13,529) for CTAS 4, and 87.0% (1,117) for CTAS 5.

Of the discharged patients, 3,566 returned to the ED within 72 hours, yielding an overall URV rate of 7.6%. Return visit rates by CTAS category were 6.7% (13/193) for CTAS 1, 7.9% (558/7,100) for CTAS 2, 8.5% (2,118/24,801) for CTAS 3, 6.0% (811/13,529) for CTAS 4, and 5.6% (62/1,117) for CTAS 5. By absolute volume, CTAS 3 accounted for the majority of return visits (2,118), followed by CTAS 4 (811) and CTAS 2 (558).

**Figure 2:**
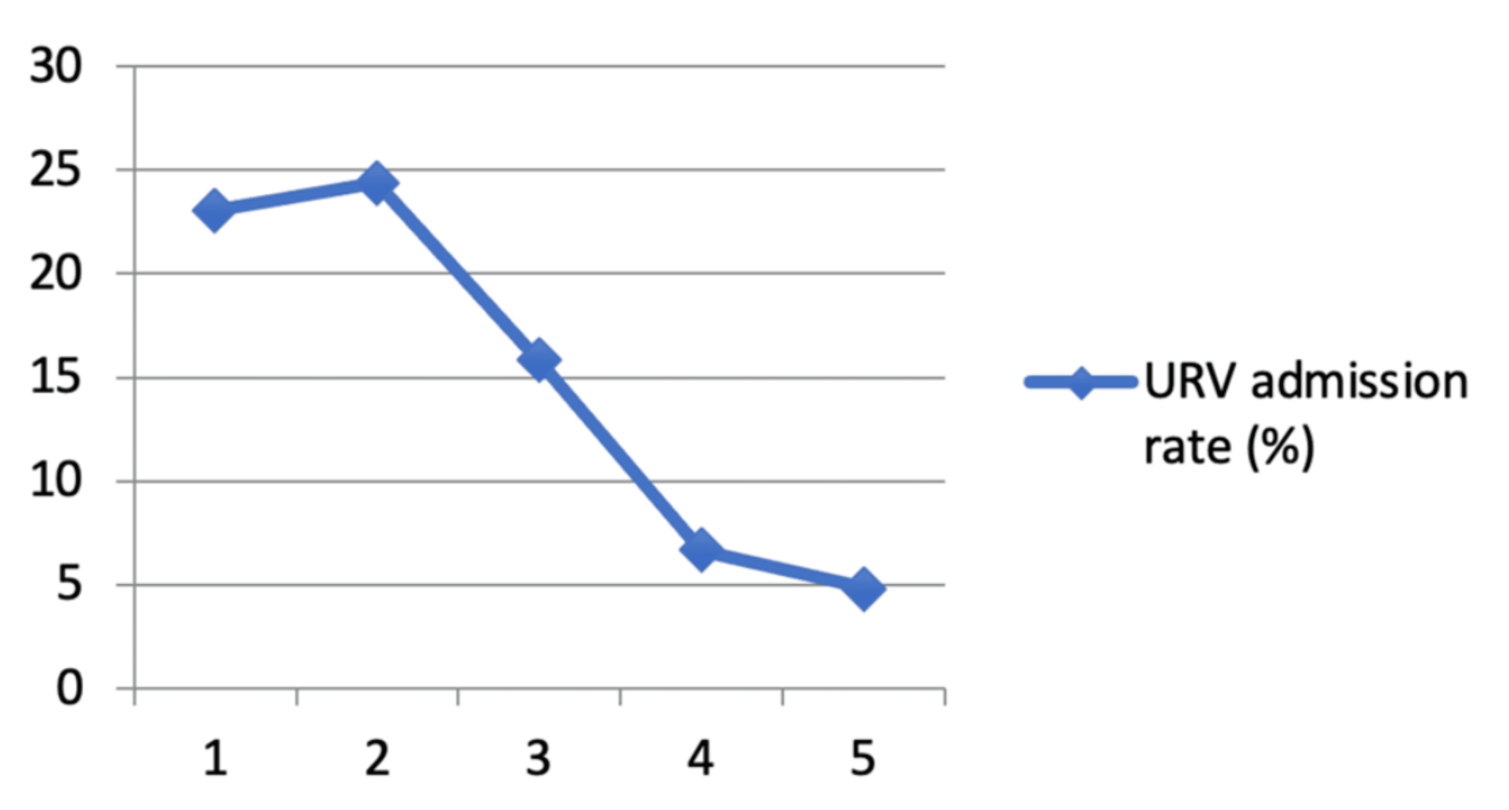
Subsequent admission rates (%) for URVs based on initial visit CTAS category CTAS: Canadian Triage and Acuity Scale; URV: unplanned return visit

Among the 3,566 URVs, 14.9% (532) resulted in hospital admission. Admission rates on return differed by initial CTAS level: 23.1% (3/13) for CTAS 1, 24.4% (136/558) for CTAS 2, 15.8% (335/2,118) for CTAS 3, 6.7% (54/811) for CTAS 4, and 4.8% (3/62) for CTAS 5 (Figure 2). In terms of volume, CTAS 3 again represented the largest group admitted on return (335), followed by CTAS 2 (136) and CTAS 4 (54), with very few admissions from CTAS 1 and 5. These results highlight both the frequency and clinical impact of return visits among CTAS 3 patients, the largest and most heterogeneous triage category.

Correlation and regression analysis

A Pearson correlation analysis showed a strong inverse relationship between CTAS level and URV admission rate (r = -0.89; 95% CI -0.99 to -0.03; R² = 0.79; F = 11.25; p = 0.04). This suggests that lower CTAS numbers (i.e., higher acuity) are associated with a higher likelihood of admission on return.

**Figure 3:**
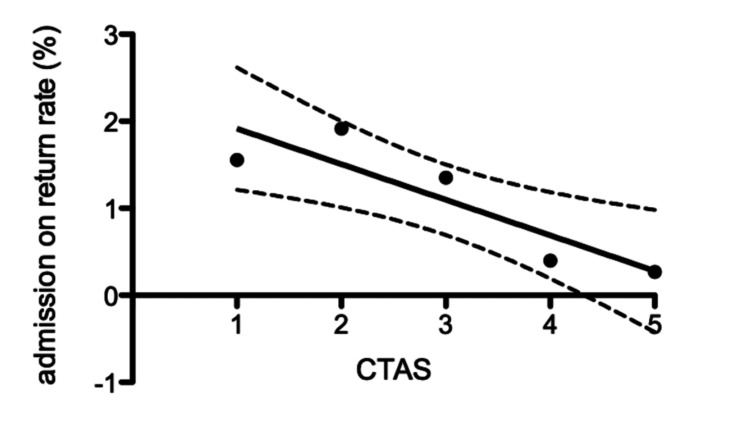
Rate of admission on return was negatively correlated with initial CTAS level (Pearson r = -0.89 (95 CI -0.99 to -0.03); R² = 0.79; F = 11.25; p = 0.04) CTAS: Canadian Triage and Acuity Scale

To further evaluate this trend, a linear regression was performed, treating CTAS level as an ordinal variable (1-5). This analysis confirmed a statistically significant inverse association (R² = 0.90, p = 0.014), with each one-point increase in CTAS level corresponding to an absolute 5.4% decrease in admission rate on return (Figure 3). These findings support the predictive value of initial triage acuity and reinforce the importance of reporting URV data by individual CTAS categories.

RR of admission

To further assess the relationship between initial triage acuity and the likelihood of admission following an unplanned return, we compared high-acuity patients (CTAS 1-2) to those triaged as CTAS 3, 4, or 5. Patients in the high-acuity group had a significantly increased risk of admission on return, with an RR of 1.90 (95% CI: 1.57 to 2.30, p < 0.0001).

## Discussion

The findings of this study demonstrate a clear relationship between initial CTAS category and subsequent URV admission rates. This aligns with the hypothesis that higher-acuity patients who are discharged have a greater likelihood of requiring subsequent admission, potentially reflecting incomplete resolution of the initial condition or the need for further investigation.

Although we report RR estimates using CTAS 4 as the reference category, we acknowledge that CTAS 3 patients may differ substantially in clinical presentation and risk profile from CTAS 4 and 5. Therefore, we present individual CTAS-level analyses throughout to allow clinicians and researchers to interpret each category independently.

Clinical implications

The relationship between initial CTAS acuity and URV admission rates carries significant clinical implications. Patients being initially triaged as CTAS 1 or 2 - representing the most urgent and potentially severe ED cases - should prompt clinicians to prioritize thorough diagnostic evaluation and ensure comprehensive discharge planning. High-acuity patients are often at greater risk of adverse outcomes, necessitating targeted follow-up strategies to mitigate the likelihood of deterioration [1,7].

CTAS 3 patients warrant special attention given their volume and clinical variability. They are often considered the “gray zone” of ED triage - urgent but not emergent - and may present with evolving conditions that are difficult to risk-stratify at the initial visit. In our analysis, they accounted for the highest absolute number of return visits and admissions, though their admission rate was lower than CTAS 1-2 and higher than CTAS 4-5. This pattern suggests that CTAS 3 patients may benefit from targeted follow-up strategies or discharge planning interventions to reduce return admissions.

From a quality improvement perspective, stratifying URV data by CTAS category offers a valuable tool for evaluating ED performance. This approach allows healthcare teams to identify patterns of care that may contribute to unplanned returns, facilitating interventions aimed at enhancing patient safety and care continuity [8-10].

Resource allocation is another critical consideration. By identifying high-risk groups based on initial triage acuity, hospitals can direct resources toward these populations, such as implementing specialized follow-up clinics or enhanced discharge protocols for those who are not admitted initially. This targeted approach not only optimizes patient outcomes but also could reduce the strain on ED resources by providing a safety net to help facilitate avoidable admissions.

Comparison with existing literature

Our results are consistent with international studies emphasizing the importance of initial triage acuity in predicting ED outcomes. For example, Hayward et al. highlighted similar trends in adult URVs [7], while Health Quality Ontario’s report underscored the value of URV metrics in evaluating care quality [8].

Limitations

This study’s single-center design may limit generalizability. Our universal government-funded healthcare system and differences in access to primary care may influence the number of visits. We have made the assumption that the unplanned return admissions are a result of a clinical need for admission; however, it is possible that some were admitted as a result of physician caution following the return. Additionally, excluding planned return visits may underestimate the true URV burden. Future research should explore the impact of other variables, such as comorbidities and social determinants, on URV outcomes.

Finally, while admission on return visit was used as a measurable outcome, it should be interpreted as a surrogate marker of clinical significance. Admission decisions may be influenced by factors beyond medical necessity, including provider caution or institutional protocols, particularly in cases where patients return with unresolved but unchanged symptoms.

## Conclusions

The initial CTAS category is a significant predictor of admission rates among patients with unplanned ED return visits. These findings reinforce the importance of detailed triage assessments, the predictive role of initial acuity in discharge decision-making, and the need for targeted quality improvement efforts such as stratified reporting of URV data by triage category to enhance patient care. Patients triaged as CTAS 1 and 2 had the highest rates of admission on return, highlighting the risks associated with early discharge in high-acuity presentations. In contrast, CTAS 3 patients accounted for the greatest number of return visits and subsequent admissions, emphasizing their importance as a focus for volume-based interventions and follow-up strategies.
